# Supplementary material for: Photonic Weyl point in a two-dimensional resonator lattice with a synthetic frequency dimension
Source: Nat Commun. 2016 Dec 15;7:13731. doi: 10.1038/ncomms13731 (PMC5172232; doi:10.1038/ncomms13731)
Supplement: Supplementary Information — Supplementary Figures, Supplementary Notes and Supplementary References. [file ncomms13731-s1.pdf]

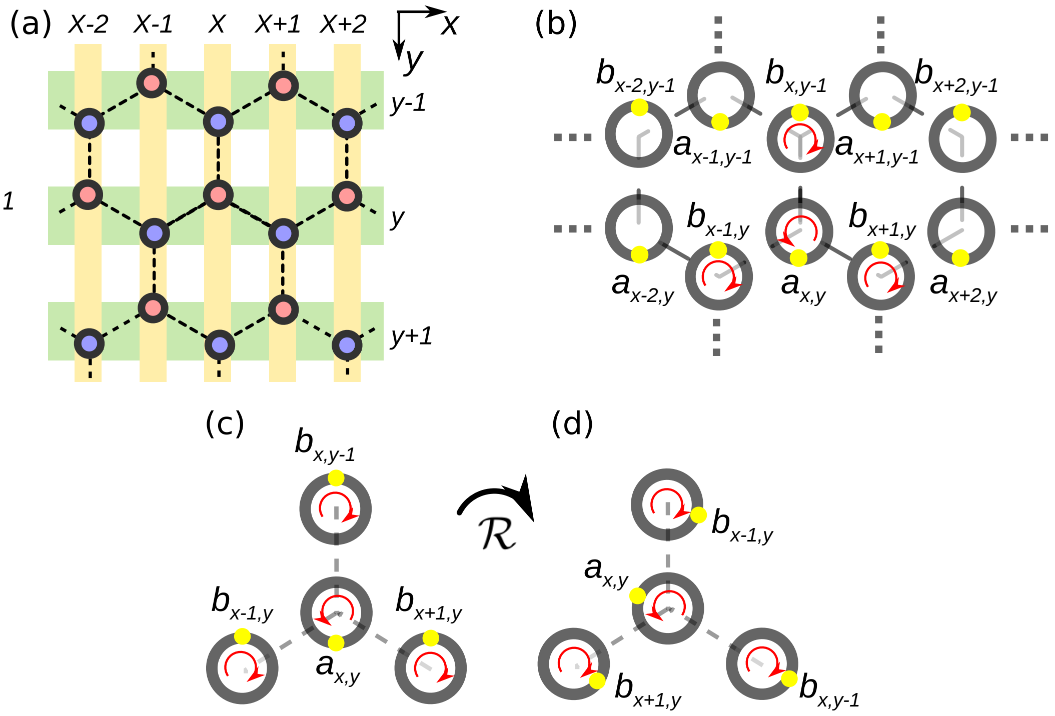

Supplementary Figure 1. Coordinate system and labelling of a honeycomb array of ring resonators. (a) The (x, y) position labelling convention for the honeycomb lattice. Red and blue dots represent resonators in the A and B sublattices, respectively. Each green bar denotes a row with same the y label, and each yellow bar denotes a column with the same x label. (b) An infinite honeycomb lattice of ring resonators. (c-d) Part of a honeycomb lattice of rings under 120° rotation around the central resonator at (x, y). Red arrows are mode circulating directions. Yellow dots represent electro-optic modulators (EOM).

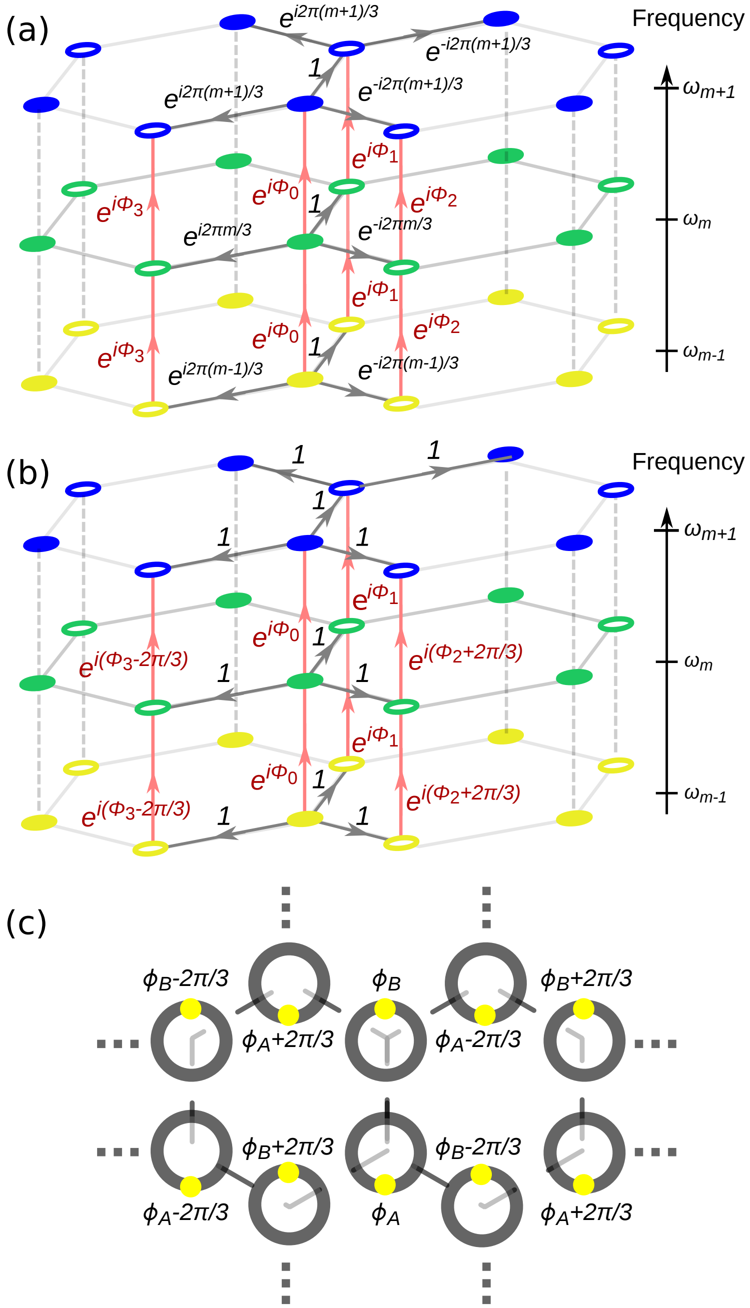

Supplementary Figure 2. Coupling phase in the 3D tight binding model. (a) Coupling phases of the tight binding model of Supplementary Equation 13. m labels the mode order. ϕ_i is the modulation phase at the ith ring. (b) Effective coupling phases after applying a gauge transformation to generate the tight binding Hamiltonian of Supplementary Equation 15. Closed and open dots represent resonators in the A and B sublattices, respectively. Yellow, green and blue represent three adjacent resonator frequencies. Red arrows denote the coupling along the synthetic frequency dimension, and grey arrows denote the coupling along spatial dimensions. (c) Modulation phases in an array of ring resonators. Non-uniform modulation phases in each sublattice are needed to achieve the Hamiltonian in Eq. 2 of the main text, where effective dynamic coupling phases are uniform on each of the A and B sublattice. The yellow dots represent EOMs.

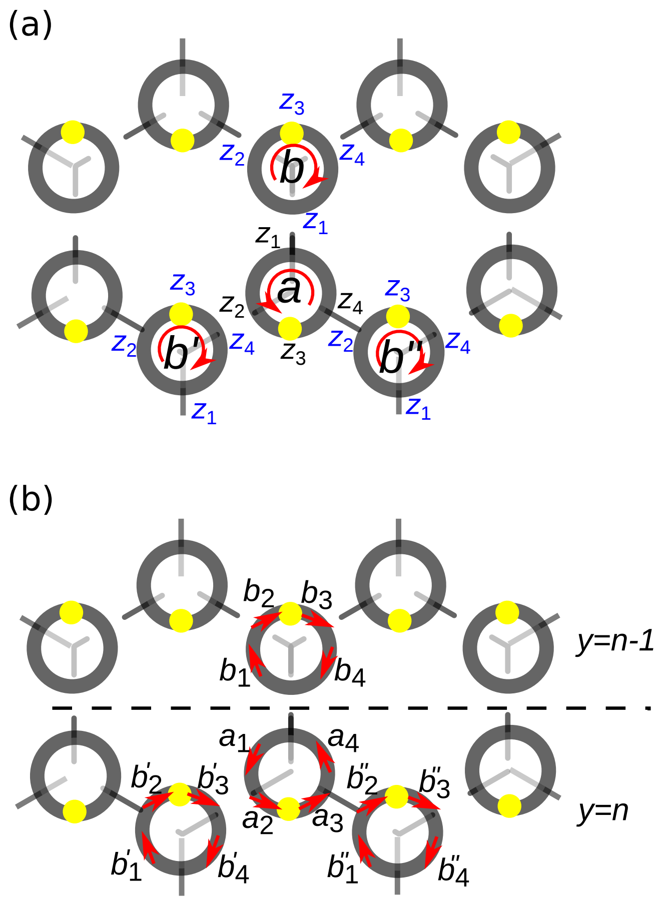

Supplementary Figure 3. Labelling variables on the ring. (a) Labels of various positions on the rings for waveguide amplitude model. Black z_i's label the positions on a ring in the A sublattice. Blue z_i's label the positions on a ring in the B sublattice. $z_{1,2,4}$ denote positions for static coupling to neighbouring rings. z_3 denotes position of an EOM. (b) Labels of the time-independent modal amplitude. $a(b)_{j,m}$'s are used for the steady state waveguide amplitude model. $a(b)_1$ is defined at the centre of z_1 and z_2. $a(b)_4$ is defined at the centre of z_1 and z_4. $a(b)_{2,3}$ are defined right before and after the EOM at z_3. These modal amplitudes are coupled through static coupling and dynamic modulation. Red arrows are mode circulation directions. Yellow dots represent EOMs.

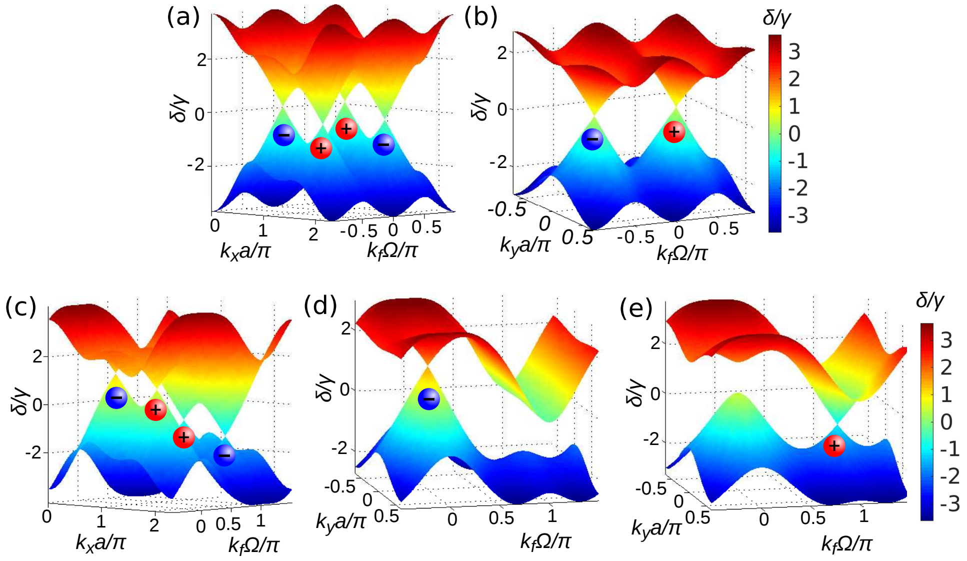

Supplementary Figure 4. Band structure for infinite systems using waveguide amplitude model. The charge of each Weyl point is lablled underneath. δ denotes frequency detuning from resonance. Electro-optic modulation depth is $\alpha = 0.65$, and inter-ring coupling is $\gamma = J_1(\alpha) = 0.3$. The existence of Weyl points and the number and charges of these Weyl points are the same as the tight binding model shown in Figs. 2 and 3 in the main text.

(a,b) Inversion symmetry breaking with modulation phases $\phi_A = 0$ and $\phi_B = \pi$. The Weyl points are located at $k_y = 0$, $k_x = \frac{4\pi}{3\sqrt{3}a}$ or $\frac{8\pi}{3\sqrt{3}a}$, and $k_f = \pm\frac{\pi}{2\Omega}$, same as in the tight binding model. (a) The band structure in $k_x - k_f$ plane at $k_y = 0$. (b) The band structure in $k_y - k_f$ plane at $k_x = \frac{4\pi}{3\sqrt{3}a}$. Unlike in the tight binding model where all Weyl points have frequency $\delta = 0$, the waveguide amplitude model predicts $\delta = \pm 0.27\gamma$ for the Weyl points at $k_x = \frac{4\pi}{3\sqrt{3}a}$ and $\frac{8\pi}{3\sqrt{3}a}$, respectively.

(c-e) Time-reversal symmetry breaking with modulation phases $\phi_A = -\phi_B = \pi/3$. The Weyl points are located at $k_y = 0$, $(k_x, k_f) \in \{(0.69\frac{\pi}{a}, 0), (1.46\frac{\pi}{a}, 0), (0.85\frac{\pi}{a}, \frac{\pi}{\Omega}), (1.62\frac{\pi}{a}, \frac{\pi}{\Omega})\}$ with $\delta = [1.33, 0.78, -0.78, -1.33]\gamma$, respectively. This differs from the tight binding model, in which the first pair of Weyl points has frequency $\delta = t$ and the second pair has $\delta = -t$. (c) The band structure in the $k_x - k_f$ plane at $k_y = 0$. (d) The band structure in the $k_y - k_f$ plane at $k_x = 0.69\frac{\pi}{a}$. (e) The band structure in the $k_y - k_f$ plane at $k_x = 0.85\frac{\pi}{a}$. The Bloch wavevectors for the Weyl points are different from the tight binding model in which both Weyl points at $k_f = 0$ and $\frac{\pi}{\Omega}$ occurs at $k_x = \frac{4\pi}{3\sqrt{3}a} \approx 0.77\frac{\pi}{a}$.

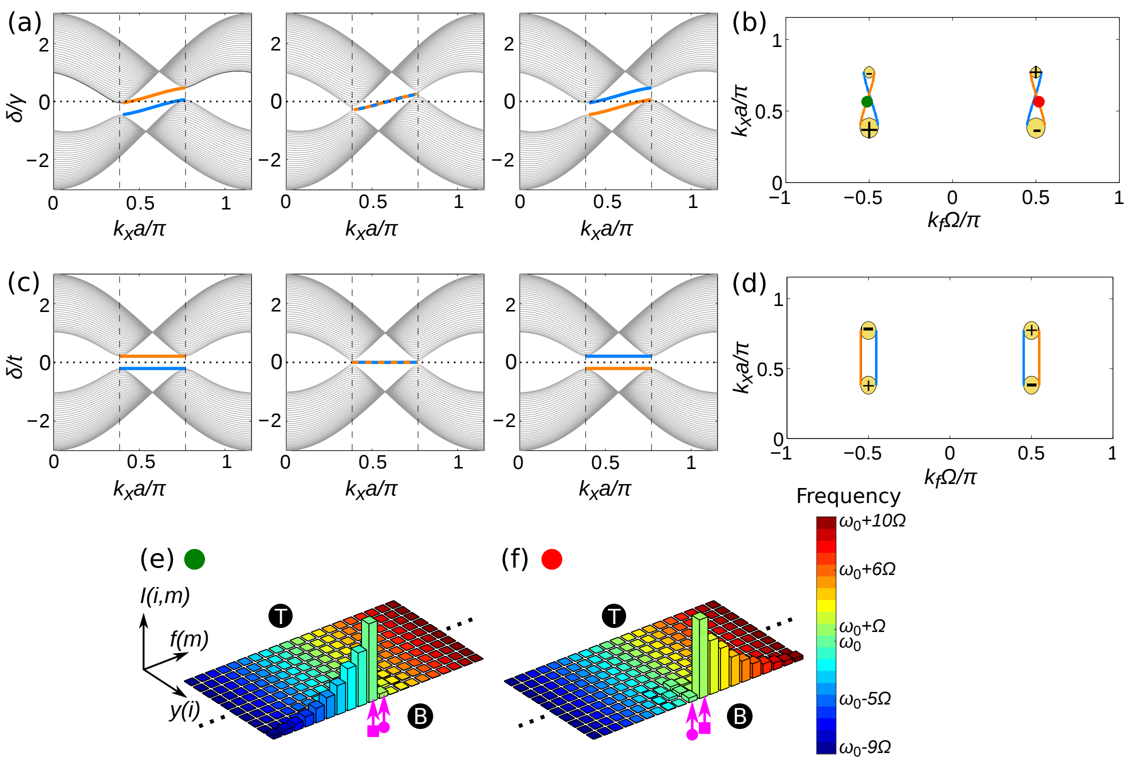

Supplementary Figure 5. A stripe with broken inversion symmetry. Modulation phases are $\phi_A = 0$ and $\phi_B = \pi$. (a,b) Band structure calculated by the waveguide amplitude model. (a) Constant k_f cuts at $k_f = (-0.53, -0.5, -0.47)\frac{\pi}{\Omega}$. (b) Constant frequency cut at $\delta = 0.07\gamma$. (c,d) Band structure calculated by the tight binding model with coupling constant t. (c) Constant k_f cuts at $k_f = (-0.53, -0.5, -0.47)\frac{\pi}{\Omega}$. (d) Constant frequency cut at $\delta = 0.3\gamma$. 40 rows in y are used. Grey lines show bulk states. Orange and blue lines show surface states of the bottom and top edges, respectively. Dashed lines show $k_x = \frac{2\pi}{3\sqrt{3}a}$ and $\frac{4\pi}{3\sqrt{3}a}$, where the Weyl points occur. These differ from the Weyl points in the bulk band structure due to Brillouin Zone folding. The waveguide amplitude model and the tight binding model both predict surface state arcs connecting a pair of Weyl points at $k_f = \pm\frac{\pi}{2\Omega}$. They also predict the same charge and position of the Weyl points in the wavevector space.

The two models also have a few differences. In the waveguide amplitude model, the Weyl point near $k_x = \frac{2\pi}{3\sqrt{3}a}$ has lower frequency than the ones near $k_x = \frac{4\pi}{3\sqrt{3}a}$. As a result, the surface states now have positive group velocity along x, which is evident by the tilt of the surface arcs in (a). On the contrary, the tight binding model predicts zero group velocity along x since the two Weyl points have the same frequency, as shown in (c). The frequency difference of the Weyl points in the waveguide amplitude model also means that if one considers a constant frequency cut between the frequencies of the Weyl points, the surface state arcs connecting the two regions of bulk states cross in the wavevector space, as shown in (b). Here the surface state arcs connect the bulk band above the Weyl point near $k_x = \frac{2\pi}{3\sqrt{3}a}$ to that below the Weyl point near $k_x = \frac{4\pi}{3\sqrt{3}a}$. If we take a constant frequency cut above both Weyl points as shown in Fig. 4c in the main text, the region of bulk states around $k_x = \frac{2\pi}{3\sqrt{3}a}$ would be larger than that around $k_x = \frac{4\pi}{3\sqrt{3}a}$. On the contrary, in the tight binding model, since the Weyl points have the same frequency, any constant frequency cut intersects either the bulk band above the Weyl points or below. As shown in (d), the surface state arcs remain parallel, and the two regions of bulk states have the same size.

(e,f) Surface state excitation on a stripe with 5 rows using the waveguide amplitude model. Calculated with $\delta = 0$, $k_x = \frac{\pi}{\sqrt{3}a}$ and 60 sidebands. Only 20 frequencies closest to the excitation are shown. A and B sites are plotted separately along y axis. Input frequencies are marked by pink arrows, with a phase difference of $\pi/2$ between square-ended and circular-ended arrows. (e) Excited with $S_{M_0+1}/S_{M_0} = -i$ to couple to $k_f = -\frac{\pi}{2\Omega}$ modes, indicated by the green dot in (b). (f) Excited with $S_{M_0+1}/S_{M_0} = i$ to couple to $k_f = \frac{\pi}{2\Omega}$ modes, indicated by the red dot in (b). Both are excited from the bottom edge B rings. Other parameters are: modulation strength $\alpha = 0.65$, inter-ring coupling $\gamma = J_1(\alpha) = 0.3$, round-trip transmission $r = 0.9$ and waveguide-ring coupling $\eta = 0.1$.

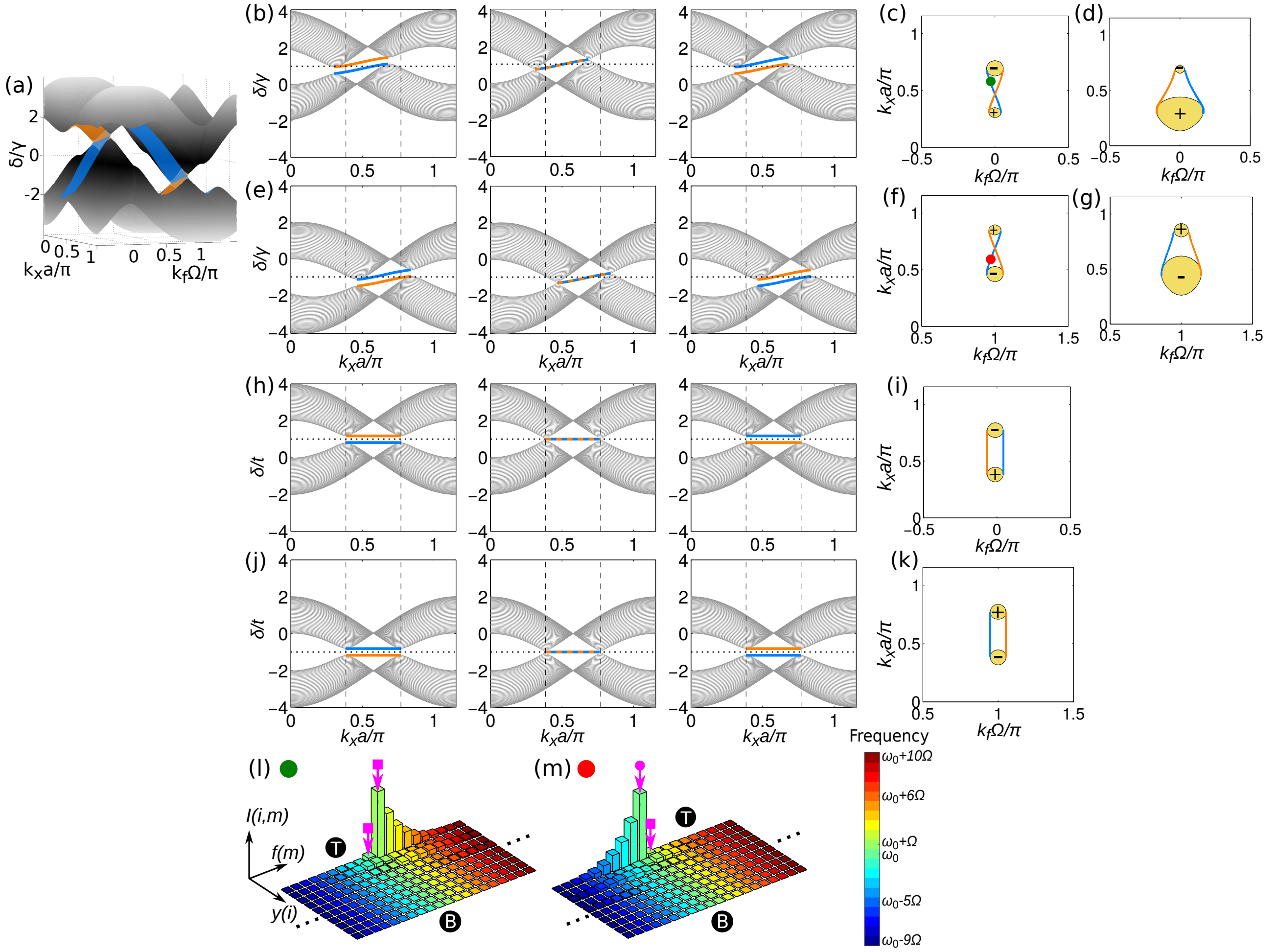

Supplementary Figure 6. A stripe with broken time-reversal symmetry. Modulation phases are $\phi_A = -\phi_B = \frac{\pi}{3}$. (a-g) Waveguide amplitude model. (a) Projected band structure showing the bulk states (grey) and the surface states (orange and blue sheets). Weyl points near $k_f = 0$ have $\delta \approx \gamma$ while those near $k_f = \frac{\pi}{\Omega}$ have $\delta \approx -\gamma$. (b) Constant k_f cuts at $k_f = (-0.03, 0, 0.03)\frac{\pi}{\Omega}$. Dotted lines show $\delta = \gamma$. (c,d) Constant frequency cut near $k_f = 0$ at $\delta = \gamma$ and 1.5γ, respectively. (e) Constant k_f cuts at $k_f = (0.97, 1, 1.03)\frac{\pi}{\Omega}$. Dotted line shows $\delta = -\gamma$. (f,g) Constant frequency cut near $k_f = \frac{\pi}{\Omega}$ at $\delta = -\gamma$ and -0.6γ, respectively. (h-k) Tight binding model with coupling constant t. (h) Constant k_f cuts at $k_f = (-0.03, 0, 0.03)\frac{\pi}{\Omega}$. Dotted line shows $\delta = t$. (i) Constant frequency cut at $\delta = 1.3t$ near $k_f = 0$. (j) Constant k_f cuts at $k_f = (0.97, 1, 1.03)\frac{\pi}{\Omega}$. Dotted line shows $\delta = -t$. (k) Constant frequency cut $\delta = -0.7t$ near $k_f = \frac{\pi}{\Omega}$. 40 rows in y are used. Grey lines show bulk states. Orange and blue lines show surface states of the bottom and top edges, respectively. Dashed lines show $k_x = \frac{2\pi}{3\sqrt{3}a}$ and $\frac{4\pi}{3\sqrt{3}a}$.

In the waveguide amplitude model, the frequency of a Weyl point near $k_x = \frac{2\pi}{3\sqrt{3}a}$ are lower than that near $k_x = \frac{4\pi}{3\sqrt{3}a}$ with the same k_f, as shown in (b,e). On the contrary, the tight binding model in (h,j) predicts equal frequency for the pair of Weyl points. Such a frequency difference implies, in the waveguide amplitude model, the non-zero group velocity along x of the surface states [(b,e) vs. (h,j)], the crossing of the surface state arcs in the wavevector space in a constant frequency cut at a frequency between the frequencies of the Weyl points [(c,f) vs. (i,k)], and the unequal size of bulk states in the constant frequency cut above both Weyl points [(d,g) vs. (i,k)]. In addition, the pair of Weyl points at $k_f = 0$ is shifted to smaller k_x, while that at $k_f = \frac{\pi}{\Omega}$ is shifted to larger k_x, as shown in (b,e). This means that the k_x for the surface state with optimal confinement is different near $k_f = 0$ and $k_f = \frac{\pi}{\Omega}$.

(l,m) Surface state excitation on a stripe with 5 rows using the waveguide amplitude model. Calculated with $k_x = \frac{\pi}{\sqrt{3}a}$ and 60 sidebands. Only 20 frequencies closest to the excitation are shown. A and B sites are plotted separately along y axis. Input frequencies as marked by pink arrows, with a phase difference of π between the square-ended and the circular-ended arrows. (l) Excited with $S_{M_0}/S_{M_0+1} = 1$, $\delta = \gamma$ to couple to $k_f \approx 0$ modes, indicated by the green dot in (e). (m) Excited with $S_{M_0}/S_{M_0+1} = -1$, $\delta = -\gamma =$ to couple to $k_f \approx \frac{\pi}{\Omega}$ modes, indicated by the red dot in (f). Both are excited from the top edge A rings. Other parameters are: modulation strength $\alpha = 0.65$, inter-ring coupling $\gamma = J_1(\alpha) = 0.3$, round-trip transmission $r = 0.9$ and waveguide-ring coupling $\eta = 0.1$.

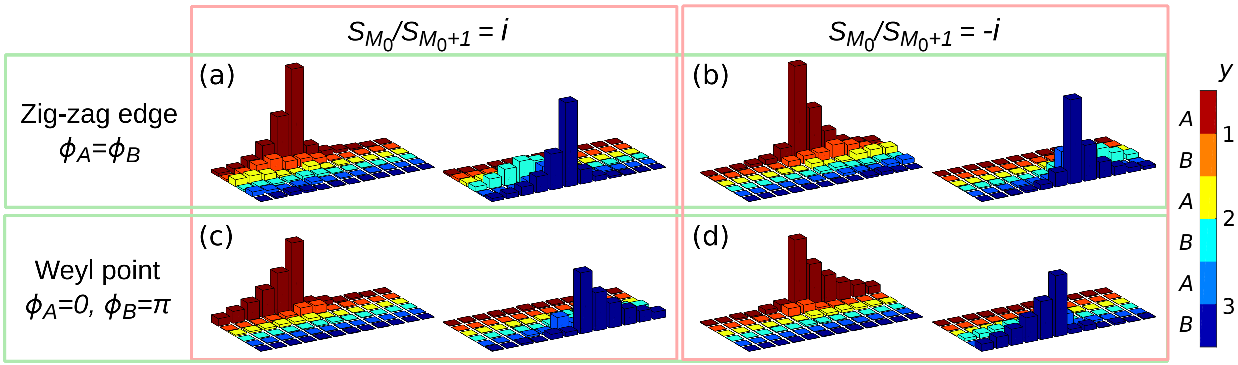

Supplementary Figure 7. Comparison between trivial and topological edge states. (a,b) On a two-dimension strip of honeycomb lattice, the zig-zag edge also supports non-chiral edge states. In our system, setting the sublattice modulation phases equal, e.i. $\phi_A = \phi_B$, corresponds to an effective three-dimensional lattice without a Weyl point, which supports edge states with the same origin as the zig-zag edge states. However, in this system the edge states on the opposite edges have the same group velocity, and thus the strip as a whole does not support chiral propagating edge states. When excited with the same input, e.g. $S_{M_0+1}/S_{M_0} = i$, the top and bottom edge states would both have frequency down-conversion. (c,d) In comparison, in a system with a Weyl point, as for example created by setting $\phi_A = 0$, $\phi_B = \pi$ in order to break the inversion symmetry, the same input will result in frequency down-conversion on the top edge and frequency up-conversion on the bottom edge. Besides the obvious change in direction of frequency conversion of the bottom edge states, the trivial zig-zag edge state also exhibits worse edge confinement and decays faster over frequencies as compared to the Weyl point edge states. Simulated for a 4×3 ring array with excitation detuning $\delta = 0$. Different colours indicate different y positions of resonators. Other parameters are: modulation strength $\alpha = 0.65$, inter-ring coupling $\gamma = J_1(\alpha) = 0.3$, round-trip transmission $r = 0.9$ and waveguide-ring coupling $\eta = 0.1$.

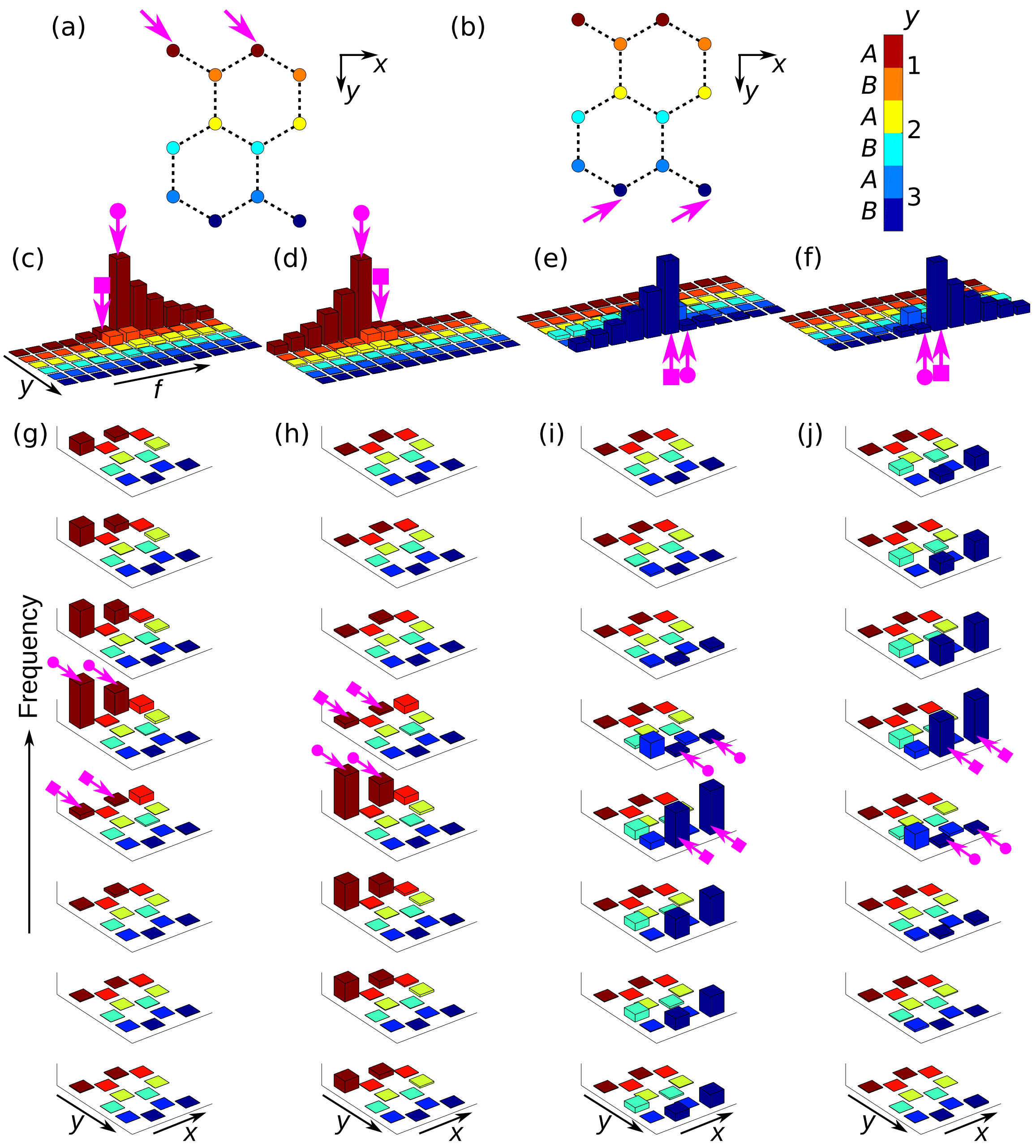

Supplementary Figure 8. Surface state transport simulation in a 4×3 array of ring resonators. (a,b) The array geometry. Each dot represents a ring resonator. Rings that have the same y coordinate are represented in the same colour. Arrows point to the rings where excitations are injected. (c-f) Intensity at different y positions and frequencies, summing over all resonators with the same y position. Input frequencies are marked by pink arrows, with a phase difference of $\pi/2$ between square-ended and circular-ended arrows. Different colours indicate different y positions of resonators, using the same encoding as (a,b). (c-d) Exciting the rings on the top edge, as shown in (a). (e-f) Exciting the rings on the bottom edge, as shown in (b). (g-j) Intensity in each ring at several frequencies adjacent to the excitation frequencies marked by pink arrows. (g-j) correspond to the same excitations as (c-f), respectively. Other parameters are: modulation strength $\alpha = 0.65$, inter-ring coupling $\gamma = J_1(\alpha) = 0.3$, round-trip transmission $r = 0.9$ and waveguide-ring coupling $\eta = 0.1$.

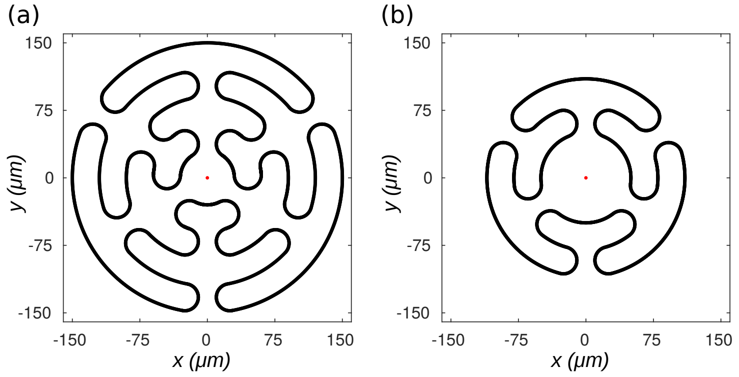

Supplementary Figure 9. Folding of a ring resonator to reduce device footprint. The shape is chosen over simple spirals as in [10] in order to preserve the three-fold rotational symmetry of the lattice. (a) A folded ring of radius 150 μm having an equivalent length of a 445 μm ring. (b) A folded ring of radius 110 μm having an equivalent length of a 230 μm ring. The minimum bending radius of both structures is 15 μm, thus introducing minimum waveguide bending loss. The minimum distance between different parts of the structure is 20 μm, making inter-waveguide coupling negligible, and carrier doping for the electrical modulation region feasible. Other parameters are: modulation strength $\alpha = 0.65$, inter-ring coupling $\gamma = J_1(\alpha) = 0.3$, round-trip transmission $r = 0.9$ and waveguide-ring coupling $\eta = 0.1$.

FSR	26 GHz	50 GHz
Ring radius R	445 μm [4]	230 μm
Array size (4×3)	$3.18{\times}3.97$ mm^2	$1.65{\times}2.07$ mm^2
Reduced ring radius (Supplementary Figure 9)	150 μm	110 μm
Reduced array size (4×3)	$1.08{\times}1.34$ mm^2	$0.79{\times}0.99$ mm^2
Coupling constant t_f	8 GHz	12 GHz
Modulation strength [5, 6]	0.5 rad mm^{-1}	
Modulation depth	0.31 rad	0.24 rad
Modulation length L_{mod}	625 μm	485 μm
Modulated length ratio $L_{\mathrm{mod}}/(2\pi R)$	0.23	0.34
Absorption loss [5, 6]	1 dB mm^{-1}	
Waveguide scattering loss [4]	0.1 dB mm^{-1}	
Round-trip loss κ_{in}	2.7 GHz	3.6 GHz
Round-trip power transmission	81.3%	86.5%
Round-trip amplitude transmission	90.1%	93.0%
Quality factor	2.4×10^5	1.7×10^5

Supplementary Table 1. Two designs for silicon ring modulator based system. Given a FSR determined by the modulation bandwidth, we choose a coupling constant t_f that is significantly larger than loss but still preserves the validity of the rotating wave approximation. The inter-ring coupling t_{xy} should match t_f by adjusting the waveguide distance and coupling region length. FSR determines the ring size. t_f and the achievable modulation strength determine the modulation region length. The modulation length ratio shows that in both cases, less then 1/3 of the ring needs to be dynamically modulated to achieve the desired coupling constant. The ring size and modulation region length determine the round-trip transmission. The round-trip transmission and the operating wavelength, assumed to be 1550 nm, determine the quality factor Q. The FSR, the modulation strength and the loss value are taken from state-of-the-art published results on depletion-mode silicon waveguide modulators [4–6].

Supplementary Note 1. Tight Binding Model for Honeycomb Lattices of Ring Resonators

In this section we will show a ring-resonator-based system that implements the Hamiltonian in Eq. 1 of the main text. We also prove the validity of the tight binding model in Eq. 2 of the main text in describing such a ring resonator lattice.

Consider the infinite honeycomb lattice of rings shown in Supplementary Figure 1a. The red and blue dots represent the centre of rings for the A and B sublattice, respectively. The dashed lines represent evanescent coupling between adjacent rings.

Supplementary Figure 1a introduces a coordinate system (x, y) for labelling the rings. Each horizontal zig-zag chain of rings, which we will called a row, is lablled with the same y. Within each row, the resonators are labelled with increasing x. As such, each integer pair (x, y) labels a resonator. All resonators in the A sublattice have indices x, y summing to an even integer, while those in the B sublattice have indices x, y summing to an odd integer. A general form of tight binding Hamiltonian describing such a honeycomb lattice is:

$$\mathcal{H} = \sum_i \omega_i c_i^\dagger c_i + \sum_{<ij>} \left(t_{ij} c_j^\dagger c_i + c.c. \right) \tag{1}$$

where $i, j \in \{(x, y)\}$ labels the position of each lattice site, c and $c^\dagger$ are the standard ladder operators, and $c.c.$ denotes complex conjugate. The second terms sums over all neighbouring pairs of i, j. In Supplementary Equation 1, each site supports a single resonant mode with a frequency ω_i, as described by the first term. The second term represents coupling between all adjacent sites, i.e. pairs of sites connected by the dashed line in Supplementary Figure 1a. Rewriting Supplementary Equation 1 in the explicit (x, y) label, we obtain:

$$\mathcal{H} = \sum_{x,y} \omega_i c_{x,y}^\dagger c_{x,y} + \sum_{x,y} \left(t_{(x,y)\to(x+1,y)} c_{x+1,y}^\dagger c_{x,y} + c.c. \right) + \sum_{x,y|x+y\in\text{odd}} \left(t_{(x,y)\to(x,y+1)} c_{x,y+1}^\dagger c_{x,y} + c.c. \right) \tag{2}$$

The second and third terms represent coupling in horizontal and vertical links, respectively. The horizontal coupling terms between x and $x+1$ column sum over all rows. The vertical coupling between y and $y+1$ rows, however, occurs only over every other column.

We now use the Hamiltonian of Supplementary Equation 1 to describe the static coupling among the m-th resonant modes of the rings in the lattice shown in Supplementary Figure 1b. For such a ring resonator lattice, the coupling is between the counter-clockwise rotating states on the A sublattice and the clockwise rotating states on the B sublattice. We label the m-th resonant modes on the A and B sublattices as $|a_{x,y,m}\rangle$ and $|b_{x,y,m}\rangle$, where $x + y$ is odd or even, respectively. Denote $|a(b)_{x,y,m}\rangle = c_{x,y,m}^\dagger |\mathbf{0}\rangle$. Under rotation of an angle θ around the centre of a resonator, its resonant modes transform as:

$$\mathcal{R}_\theta^{(x,y)} |a_{x,y,m}\rangle = e^{-im\theta} |a_{x,y,m}\rangle, \ \mathcal{R}_\theta^{(x,y)} |b_{x,y,m}\rangle = e^{im\theta} |b_{x,y,m}\rangle \tag{3}$$

where the subscript of the rotation operator $\mathcal{R}$ represents the angle of rotation, and the superscript represents the centre of rotation.

Using the basis of the m-th resonant modes, the static coupling among these modes is described by:

$$\mathcal{H}_m = \omega_m \sum_i |a(b)_{i,m}\rangle \langle a(b)_{i,m}| + t_{xy} \sum_{<ij>} \left(e^{i\varphi_{ij}} |b_{j,m}\rangle \langle a_{i,m}| + c.c. \right) \tag{4}$$

Below we will constrain the coupling constants by the rotational symmetry of the structure.

Consider the terms in $\mathcal{H}_m$ related to the coupling terms involving one particular resonator in the A sublattice labelled as (x, y), as schematically shown in Supplementary Figure 1c:

$$\mathcal{H}_m = ... + (t_1 |b_{x,y-1,m}\rangle \langle a_{x,y,m}| + t_2 |b_{x+1,y,m}\rangle \langle a_{x,y,m}| + t_3 |b_{x-1,y,m}\rangle \langle a_{x,y,m}| + c.c.) + ... \tag{5}$$

The system is invariant under 120° rotation around the centre of the resonator at (x, y), denoted by operator $\mathcal{R} = \mathcal{R}_{\pi/3}^{(x,y)}$, i.e.,

$$\mathcal{R}\mathcal{H}_m\mathcal{R}^\dagger = \mathcal{H}_m \tag{6}$$

On the other hand, from Supplementary Figures 1c,d we can deduce that

$$\begin{gathered} \mathcal{R} |a_{x,y,m}\rangle = e^{-i\frac{2\pi m}{3}} |a_{x,y,m}\rangle \\ \mathcal{R} |b_{x,y-1,m}\rangle = e^{i\frac{2\pi m}{3}} |b_{x+1,y,m}\rangle, \ \mathcal{R} |b_{x+1,y,m}\rangle = e^{i\frac{2\pi m}{3}} |b_{x-1,y,m}\rangle, \ \mathcal{R} |b_{x-1,y,m}\rangle = e^{i\frac{2\pi m}{3}} |b_{x,y-1,m}\rangle \end{gathered} \tag{7}$$

and therefore

$$\mathcal{R}\mathcal{H}_m\mathcal{R}^\dagger = ... + e^{i\frac{4\pi m}{3}}\left(t_1 \left|b_{x+1,y,m}\right\rangle \left\langle a_{x,y,m}\right| + t_2 \left|b_{x-1,y,m}\right\rangle \left\langle a_{x,y,m}\right| + t_3 \left|b_{x,y-1,m}\right\rangle \left\langle a_{x,y,m}\right| + c.c.\right) + ... \tag{8}$$

Comparing Supplementary Equations 5-8 we obtain:

$$t_2 = e^{-i\frac{2\pi m}{3}} t_1,\ t_3 = e^{i\frac{2\pi m}{3}} t_1 \tag{9}$$

Without loss of generality we pick the phase of t_1 to be zero, i.e., $t_1 = t_{xy}$. Supplementary Equation 5 becomes

$$\mathcal{H}_m = ... + t_{xy}\Big(\left|b_{x,y-1,m}\right\rangle \left\langle a_{x,y,m}\right| + e^{-i\frac{2\pi m}{3}} \left|b_{x+1,y,m}\right\rangle \left\langle a_{x,y,m}\right| + e^{i\frac{2\pi m}{3}} \left|b_{x-1,y,m}\right\rangle \left\langle a_{x,y,m}\right| + c.c.\Big) + ... \tag{10}$$

Reverting to the second quantization form, we therefore have:

$$\mathcal{H}_m = \sum_{x,y} \omega_m c^\dagger_{x,y,m} c_{x,y,m} + t_{xy}\sum_{x,y}(e^{-i\frac{2\pi m}{3}} c^\dagger_{x+1,y,m} c_{x,y,m} + c.c.) + t_{xy}\sum_{x,y|x+y\in\text{odd}} (c^\dagger_{x,y+1,m} c^\dagger_{x,y,m} + c.c.) \tag{11}$$

We note that the coupling along x has a directional, m-dependent phase as shown in Supplementary Figure 2a.

Now consider applying an index modulation with frequency $\Omega = \omega_{m+1} - \omega_m$ to each ring to couple the different orders of resonant modes. We assume that the modulation at a ring (x, y) has a modulation phase $\phi_{x,y}$. The term in the Hamiltonian describing the effect of modulation is:

$$2t_f \sum_m \cos(\Omega t + \phi_{x,y})(c^\dagger_{x,y,m+1} c_{x,y,m} + c^\dagger_{x,y,m} c_{x,y,m+1}) \tag{12}$$

Overall, the Hamiltonian describing the honeycomb lattice of resonator under dynamic modulation is:

$$\begin{aligned}\mathcal{H} = &\sum_{x,y,m} \omega_m c^\dagger_{x,y,m} c_{x,y,m} + t_{xy}\sum_{x,y,m}(e^{-i\frac{2\pi m}{3}} c^\dagger_{x+1,y,m} c_{x,y,m} + c.c.) \\ &+ t_{xy}\sum_{x,y,m|x+y\in\text{odd}} (c^\dagger_{x,y+1,m} c^\dagger_{x,y,m} + c.c.) + 2t_f \sum_{x,y,m} \cos(\Omega t + \phi_{x,y})(c^\dagger_{x,y,m+1} c_{x,y,m} + c.c.)\end{aligned} \tag{13}$$

which describes a three-dimensional lattice lablled by (x, y, m). Performing a gauge transformation $\tilde{c}_{x,y,m} \equiv e^{imx\frac{2\pi}{3}} c_{x,y,m}$ results in:

$$\begin{aligned}\mathcal{H} = &\sum_{x,y,m} \omega_m \tilde{c}^\dagger_{x,y,m} \tilde{c}_{x,y,m} + t_{xy}\sum_{x,y,m}(\tilde{c}^\dagger_{x+1,y,m} c_{x,y,m} + c.c.) + t_{xy}\sum_{x,y,m|x+y\in\text{odd}} (\tilde{c}^\dagger_{x,y+1,m} \tilde{c}^\dagger_{x,y,m} + c.c.) \\ &+ 2t_f \sum_{x,y,m} \cos(\Omega t + \phi_{x,y})(e^{i\frac{2\pi x}{3}} \tilde{c}^\dagger_{x,y,m+1} \tilde{c}_{x,y,m} + c.c.)\end{aligned} \tag{14}$$

As a result of the gauge transformation, the static (spatial) coupling is homogeneous and m-independent. On the other hand, the coupling along the synthetic frequency dimension now has an additional x-dependent, directional phase, as shown in Supplementary Figure 2b. Further simplifying the above Hamiltonian by changing the (x, y) labelling to the $i, j \in \{(x, y)\}$ labelling for the static coupling terms, we obtain:

$$\mathcal{H} = \sum_{i,m} \omega_m \tilde{c}^\dagger_{i,m} \tilde{c}_{i,m} + t_{xy}\sum_{<ij>,m}(\tilde{c}^\dagger_{j,m} \tilde{c}_{i,m} + c.c.) + 2t_f \sum_{x,y,m} \cos(\Omega t + \phi_{x,y})(e^{i\frac{2\pi x}{3}} \tilde{c}^\dagger_{x,y,m+1} \tilde{c}_{x,y,m} + c.c.) \tag{15}$$

This Hamiltonian is very similar to Eq. 1 in the main text once we replace $\tilde{c}_{i,m}$ with $a_{i,m}$. Defining $c_{i,m} = \tilde{c}_{i,m} e^{-i\omega_m t}$ which performs a transformation to a rotating frame, and applying the rotating wave approximation to remove the time-dependency of Eq. 15, we obtain:

$$\mathcal{H} = t_{xy}\sum_{<ij>,m}(c^\dagger_{j,m} c_{i,m} + c.c.) + t_f \sum_{x,y,m}(e^{i(\phi_{x,y} + \frac{2\pi x}{3})} c^\dagger_{x,y,m+1} c_{x,y,m} + c.c.) \tag{16}$$

With a redefinition of $\phi_i = \phi_{x,y} + \frac{2\pi x}{3}$, Eq. 16 matches Eq. 2 in the main text. Therefore, the Hamiltonian of Eq. 2 in the main text can be implemented in a lattice of rings provided that the modulation phases are appropriately chosen.

Supplementary Note 2. Waveguide amplitude model

In this section, we provide a more detailed description of our system using coupled mode theory, where the dynamic variables are the position-dependent waveguide modal amplitudes in each ring. We refer to such a model as the waveguide amplitude model. Compared to the tight binding model, the waveguide amplitude model provides more microscopic details of the effect of inter-ring coupling and the dynamic index modulation. Here we first set up the general waveguide amplitude equations describing the system. In the next three sections we apply this model to calculate band structures and surface states.

In general, the electric field E inside a ring can be written as [1, 2]

$$E(t, r_\perp, z) = \sum_m \mathcal{E}_m(t, z) E(r_\perp) e^{-i\omega_m t} \tag{17}$$

where $r_\perp$ denotes the direction perpendicular to the waveguide that forms the ring, and $E(r_\perp)$ is the modal profile of the waveguide mode. z denotes the longitudinal direction of the waveguide. ω_m and $\mathcal{E}_m(t, z)$ are the resonant frequency and the modal amplitude of the m-th mode. Here we assume that the waveguide is single-moded. In addition, since the modulation frequency is typically much smaller than the optical frequency, we assume that the model profile does not change over the frequency range of interest. $\mathcal{E}_m$ is assumed to be slow-varying, which implies

$$\left(\frac{\partial}{\partial z} - i\beta(\omega_m)\right)\mathcal{E}_m + \frac{1}{v_\mathrm{g}}\frac{\partial}{\partial t}\mathcal{E}_m = 0 \tag{18}$$

where v_g is the group velocity in the waveguide.

For each ring, static coupling to its neighbouring rings occur at position z_1, z_2, z_4, as shown in Supplementary Figure 3a. The equations at each coupler are:

$$\begin{pmatrix} \mathcal{E}_m(z_4^a, t^+) \\ \mathcal{E}_m(z_2^{b''}, t^+) \end{pmatrix} = S(\gamma) \begin{pmatrix} \mathcal{E}_m(z_4^a, t^-) \\ \mathcal{E}_m(z_2^{b''}, t^-) \end{pmatrix} \tag{19}$$

$$\begin{pmatrix} \mathcal{E}_m(z_2^a, t^+) \\ \mathcal{E}_m(z_4^{b'}, t^+) \end{pmatrix} = S(\gamma) \begin{pmatrix} \mathcal{E}_m(z_2^a, t^-) \\ \mathcal{E}_m(z_4^{b'}, t^-) \end{pmatrix} \tag{20}$$

$$\begin{pmatrix} \mathcal{E}_m(z_1^a, t^+) \\ \mathcal{E}_m(z_1^{b}, t^+) \end{pmatrix} = S(\gamma) \begin{pmatrix} \mathcal{E}_m(z_1^a, t^-) \\ \mathcal{E}_m(z_1^{b}, t^-) \end{pmatrix} \tag{21}$$

where

$$S(\gamma) = \begin{pmatrix} \sqrt{1-\gamma^2} & i\gamma \\ i\gamma & \sqrt{1-\gamma^2} \end{pmatrix} \tag{22}$$

describes the coupling between two rings. γ is the coupling strength.

Dynamic coupling between modes of different order m in the same resonator is achieved by an EOM at $z_3 = (z_2 + z_4)/2$, which is modeled by [1]:

$$E(t^+, r_\perp, z_3) = e^{i\alpha\cos(\Omega t + \phi)} E(t^-, r_\perp, z_3) \tag{23}$$

Using the Jacobi-Anger expansion:

$$e^{i\alpha\cos(\Omega t+\phi)} = \sum_{q=-\infty}^{\infty} i^q J_q(\alpha) e^{iq(\Omega t + \phi)} \tag{24}$$

where $J_q(\alpha)$ is the Bessel function of the first kind, we obtain a relation of the electric field before and after modulation:

$$\begin{aligned} E(t^+, r_\perp, z_3) &= \sum_m \mathcal{E}_m(t^-, z_3) E(r_\perp) e^{-i\omega_m t} \sum_{q=-\infty}^{\infty} i^q J_q(\alpha) e^{iq(\Omega t+\phi)} \\ &= \sum_{m'} E(r_\perp) e^{-i\omega_{m'} t} \sum_m \mathcal{E}_m(t^-, z_3) i^{m-m'} J_{m-m'}(\alpha) e^{i(m-m')\phi} \end{aligned} \tag{25}$$

where $m' \equiv m - q$. Switching the labels m and m', we obtain:

$$E(t^+, r_\perp, z_3) \equiv \sum_m \mathcal{E}_m(t^+, z_3) E(r_\perp) e^{-i\omega_m t} = \sum_m E(r_\perp) e^{-i\omega_m t} \times \left(\sum_{m'} \mathcal{E}_{m'}(t^-, z_3) i^{m'-m} J_{m'-m}(\alpha) e^{i(m'-m)\phi} \right) \tag{26}$$

therefore

$$\mathcal{E}_m(t^+, z_3) = \sum_{m'} \mathcal{E}_{m'}(t^-, z_3) i^{m'-m} J_{m'-m}(\alpha) e^{i(m'-m)\phi} \tag{27}$$

which relates the modal amplitudes $\mathcal{E}_m$ for different m's before and after the modulation. Supplementary Equation 27 thus describes a long-range coupling along the synthetic frequency dimension.

In summary, the modal amplitudes evolve in each section of the ring determined by Supplementary Equation 18. Couplings between rings are described by Supplementary Equations 19-21. The dynamic modulation which couples different frequencies are described by Supplementary Equation 27. Supplementary Equations 18-27 provide a complete description of the time evolution of the fields in the system, and are the defining equations of the waveguide amplitude model.

Within each ring, the couplers to the neighbouring rings and the EOM separate the ring into four sections. Within each section, the steady-state solution to Supplementary Equation 18 has the form:

$$\mathcal{E}_m(t, z) = \mathcal{E}_m(z) e^{-i\delta\omega t} = \mathcal{E}_m(z_0) e^{i[\beta(\omega_m) + \delta\omega/v_g](z - z_0)} e^{-i\delta\omega t} \tag{28}$$

where $\delta\omega$ is a small detuning from resonant frequency, z_0 is a position in the section. Let $a(b)_{k,m}$ be the time-independent modal amplitudes $\mathcal{E}_m(z)$ defined at the points in the k-th section as shown in Supplementary Figure 3a. For $k = 1, 4$, $a(b)_{k,m}$ are at the centre of the respective sections. For $k = 2, 3$, $a(b)_{k.m}$ are immediately before and after the modulator, which is located at the midpoint between the two couplers. $\mathcal{E}_m(z)$ can be expressed using $a(b)_{j,m}$ section-wise as:

$$\mathcal{E}_m(z) = a(b)_{j,m} e^{i[\beta(\omega_m) + \delta\omega/v_g](z - z_{j0})} \tag{29}$$

Note that for a resonant mode $\beta(\omega_m)L = 2m\pi$, where L is the circumference of the ring, and $z_2 - z_1 = z_4 - z_2 = z_1 - z_4 = L/3$, as shown in Supplementary Figure 3a. Furthermore, define $\delta = (\delta\omega/v_g)L$ as the round-trip phase detuning. For convenience, in the following we will refer to δ as detuning, bearing in mind its aforementioned relation to the actual frequency detuning $\delta\omega$. Now we can re-write Supplementary Equations 19-27 using $a(b)_{j,m}$ to obtain the set of equations for the steady state modes:

$$\begin{pmatrix} \mathcal{E}_m(z_4^{a+}) \\ \mathcal{E}_m(z_2^{b''+}) \end{pmatrix} = S(\gamma) \begin{pmatrix} \mathcal{E}_m(z_4^{a-}) \\ \mathcal{E}_m(z_2^{b''-}) \end{pmatrix} \implies \begin{pmatrix} a_{4,m} \\ b''_{2,m} \end{pmatrix} = e^{i\frac{2m\pi+\delta}{3}} S(\gamma) \begin{pmatrix} a_{3,m} \\ b''_{1,m} \end{pmatrix} \tag{30}$$

$$\begin{pmatrix} \mathcal{E}_m(z_2^{a+}) \\ \mathcal{E}_m(z_4^{b'+}) \end{pmatrix} = S(\gamma) \begin{pmatrix} \mathcal{E}_m(z_2^{a-}) \\ \mathcal{E}_m(z_4^{b'-}) \end{pmatrix} \implies \begin{pmatrix} a_{2,m} \\ b'_{4,m} \end{pmatrix} = e^{i\frac{2m\pi+\delta}{3}} S(\gamma) \begin{pmatrix} a_{1,m} \\ b'_{3,m} \end{pmatrix} \tag{31}$$

$$\begin{pmatrix} \mathcal{E}_m(z_1^{a+}) \\ \mathcal{E}_m(z_1^{b+}) \end{pmatrix} = S(\gamma) \begin{pmatrix} \mathcal{E}_m(z_1^{a-}) \\ \mathcal{E}_m(z_1^{b-}) \end{pmatrix} \implies \begin{pmatrix} a_{1,m} \\ b_{1,m} \end{pmatrix} = e^{i\frac{2m\pi+\delta}{3}} S(\gamma) \begin{pmatrix} a_{4,m} \\ b_{4,m} \end{pmatrix} \tag{32}$$

$$\mathcal{E}_m(z_3^+) = \sum_{m'} \mathcal{E}_{m'}(z_3^-) i^{m'-m} J_{m'-m}(\alpha) e^{i(m'-m)\phi} \implies a(b)_{3,m} = \sum_{m'} a(b)_{2,m'} i^{m'-m} J_{m'-m}(\alpha) e^{i(m'-m)\phi} \tag{33}$$

Supplementary Equations 30-33 thus define a discrete linear system with each ring represented by the amplitudes $a(b)_{k,m}$. In the following, we will solve this discrete system to obtain band structures for the bulk and the surfaces.

Supplementary Note 3. Band Structure of an Infinite Lattice

First we solve for the band structure of a lossless, infinite system in both spatial dimensions as well as in the synthetic frequency dimension. For such a system we observe certain symmetries that will simplify the form of our solution. Supplementary Equation 33 is invariant under a transformation of $m \to m + 1$, while Supplementary

Equations 30-32 are invariant under a transformation of $m \to m+3$. Below, we will prove that a steady state solution of Supplementary Equations 30-33 satisfies:

$$a(b)_{j,m} = e^{im(k_f \Omega + \theta_j)} a(b)_{j,0} \tag{34}$$

where a particular resonance with resonant frequency ω_0 is defined as the zeroth order mode, and the m-th order mode is related to the zeroth order mode by $\omega_m = \omega_0 + m\Omega$. Supplementary Equation 34 is similar to the standard form of a Bloch wave function, but with the θ_j term introduced to account for the different translational symmetry in m between Supplementary Equation 33 and Supplementary Equations 30-32. The θ_js are determined as follows. First of all, since the entire set of equations 30-33 are invariant under the transformation of $m \to m+3$, the θ_j's should satisfy $3\theta_{j,m} = 2\pi N$. Plugging Supplementary Equation 34 into Supplementary Equation 33:

$$\begin{aligned} a(b)_{3,m} &= a(b)_{2,0} \left(\sum_{m'} e^{im'(k_f \Omega + \theta_2)} i^{m'-m} J_{m'-m}(\alpha) e^{i(m'-m)\phi_{A(B)}} \right) \\ &= e^{im(k_f \Omega + \theta_2)} a(b)_{2,0} e^{i\alpha \cos(\phi_{A(B)} + k_f \Omega + \theta_2)} = e^{im(k_f \Omega + \theta_2)} a(b)_{3,0} \end{aligned} \tag{35}$$

implies that $\theta_3 = \theta_2$. Without loss of generality we set $\theta_2 = \theta_3 = 0$, then

$$a(b)_{2,m} = e^{imk_f \Omega} a(b)_{2,0},\ a(b)_{3,m} = e^{imk_f \Omega} a(b)_{3,0} \tag{36}$$

$$a(b)_{3,m} = a(b)_{2,m} e^{i\alpha \cos(\phi_{A(B)} + k_f \Omega)} \tag{37}$$

The system also has translational symmetry in the spatial dimensions. Therefore b, b', b'' are related by:

$$b'_{j,m} = e^{i(\frac{3}{2}k_y a - \frac{\sqrt{3}}{2}k_x a)} e^{im\theta'} b_{j,m} \tag{38}$$

$$b''_{j,m} = e^{i(\frac{3}{2}k_y a + \frac{\sqrt{3}}{2}k_x a)} e^{im\theta''} b_{j,m} \tag{39}$$

as required by the Bloch theorem applied to the spatial dimensions. a is the nearest neighbour distance. We note that the θ' and θ'' are j-independent since the spatial translational symmetry applies to the ring as a whole. Here we separate the Bloch phase into two parts to provide a convenient definition of the wavevectors k_x and k_y.

By properly choosing the θ_j's, θ' and θ'', Supplementary Equations 30-32 for all m can be reduced to a self-consistent relation between $\{a_{j,0}, b_{j,0} | j = 1, 2, 3, 4\}$'s. From Supplementary Equation 30 we derive

$$\begin{pmatrix} e^{im\theta_4} a_{4,0} \\ e^{im\theta''} b_{2,0} \end{pmatrix} = e^{i\frac{2m\pi+\delta}{3}} \begin{pmatrix} 1 & 0 \\ 0 & e^{-i(\frac{3}{2}k_y a + \frac{\sqrt{3}}{2}k_x a)} \end{pmatrix} S(\gamma) \begin{pmatrix} 1 & 0 \\ 0 & e^{i(\frac{3}{2}k_y a + \frac{\sqrt{3}}{2}k_x a)} \end{pmatrix} \begin{pmatrix} a_{3,0} \\ e^{im(\theta''+\theta_1)} b_{1,0} \end{pmatrix} \tag{40}$$

$$\implies \theta_4 = \theta'' = \frac{2\pi}{3},\ \theta_1 = -\theta'' = -\frac{2\pi}{3} \tag{41}$$

and similarly from Supplementary Equations 31 and 32 we derive:

$$\theta_1 = \theta' = -\frac{2\pi}{3},\ \theta_4 = -\theta' = \frac{2\pi}{3},\ \theta_1 = \theta_4 + \frac{2\pi}{3} \tag{42}$$

therefore

$$a(b)_{1,m} = e^{-im\frac{2\pi}{3}} e^{imk_f \Omega} a(b)_{1,0},\ a(b)_{4,m} = e^{im\frac{2\pi}{3}} e^{imk_f \Omega} a(b)_{4,0} \tag{43}$$

$$b'_{j,m} = e^{i(\frac{3}{2}k_y a - \frac{\sqrt{3}}{2}k_x a)} e^{-im\frac{2\pi}{3}} b_{j,m} \tag{44}$$

$$b''_{j,m} = e^{i(\frac{3}{2}k_y a + \frac{\sqrt{3}}{2}k_x a)} e^{im\frac{2\pi}{3}} b_{j,m} \tag{45}$$

Combining Supplementary Equations 33, 36, 44 and 45 we obtain:

$$b'_{2(3),m} = e^{-im\frac{2\pi}{3}} e^{imk_f \Omega} b'_{2(3),0} \implies b'_{3,m} = b'_{2,m} e^{i\alpha \cos(\phi'_B - \frac{2\pi}{3} + k_f \Omega)} \tag{46}$$

$$b''_{2(3),m} = e^{im\frac{2\pi}{3}} e^{imk_f \Omega} b''_{2(3),0} \implies b''_{3,m} = b''_{2,m} e^{i\alpha \cos(\phi''_B + \frac{2\pi}{3} + k_f \Omega)} \tag{47}$$

By applying the choice of modulation phases as shown in Supplementary Figure 2c, we have $\phi'_B = \phi_B + \frac{2\pi}{3}$ and $\phi''_B = \phi_B - \frac{2\pi}{3}$. b, b', b'' will then have the same phase $\alpha \cos(\phi_B + k_f \Omega)$ resulting from the dynamic coupling.

In summary, the system of equations derived from Supplementary Equations 30-33 for the infinite 3D lattice is:

$$\begin{pmatrix} a_{4,0} \\ b_{2,0} \end{pmatrix} = e^{i\frac{\delta}{3}} \begin{pmatrix} 1 & 0 \\ 0 & e^{-i(\frac{3}{2}k_y a - \frac{\sqrt{3}}{2}k_x a)} \end{pmatrix} S(\gamma) \begin{pmatrix} e^{i\alpha\cos(\phi_A + k_f \Omega)} & 0 \\ 0 & e^{i(\frac{3}{2}k_y a - \frac{\sqrt{3}}{2}k_x a)} \end{pmatrix} \begin{pmatrix} a_{2,0} \\ b_{1,0} \end{pmatrix} \equiv e^{i\frac{\delta}{3}} T_1 \begin{pmatrix} a_{2,0} \\ b_{1,0} \end{pmatrix} \tag{48}$$

$$\begin{pmatrix} a_{2,0} \\ b_{4,0} \end{pmatrix} = e^{i\frac{\delta}{3}} \begin{pmatrix} 1 & 0 \\ 0 & e^{-i(\frac{3}{2}k_y a + \frac{\sqrt{3}}{2}k_x a)} \end{pmatrix} S(\gamma) \begin{pmatrix} 1 & 0 \\ 0 & e^{i(\frac{3}{2}k_y a + \frac{\sqrt{3}}{2}k_x a)} e^{i\alpha\cos(\phi_B + k_f \Omega)} \end{pmatrix} \begin{pmatrix} a_{1,0} \\ b_{2,0} \end{pmatrix} \equiv e^{i\frac{\delta}{3}} T_2 \begin{pmatrix} a_{1,0} \\ b_{2,0} \end{pmatrix} \tag{49}$$

$$\begin{pmatrix} a_{1,0} \\ b_{1,0} \end{pmatrix} = e^{i\frac{\delta}{3}} S(\gamma) \begin{pmatrix} a_{4,0} \\ b_{4,0} \end{pmatrix} \equiv e^{i\frac{\delta}{3}} T_3 \begin{pmatrix} a_{4,0} \\ b_{4,0} \end{pmatrix} \tag{50}$$

Supplementary Equations 48-50 can be formulated as an eigenvalue problem

$$T(k_x, k_y, k_f)u = e^{-i\delta/3}u \tag{51}$$

from which we can determine the band structure that relates δ to (k_x, k_y, k_f). Here:

$$u = [a_{1,0}, b_{1,0}, a_{2,0}, b_{2,0}, a_{4,0}, b_{4,0}]^T \tag{52}$$

$$T = T(k_x, k_y, k_f; \gamma, \alpha, \phi_A, \phi_B) = \begin{pmatrix} 0&0&0&0&1&0 \\ 0&0&0&0&0&1 \\ 0&0&1&0&0&0 \\ 0&1&0&0&0&0 \\ 1&0&0&0&0&0 \\ 0&0&0&1&0&0 \end{pmatrix} \cdot \begin{pmatrix} T_1 & \mathbf{0} & \mathbf{0} \\ \mathbf{0} & T_2 & \mathbf{0} \\ \mathbf{0} & \mathbf{0} & T_3 \end{pmatrix} \cdot \begin{pmatrix} 0&0&1&0&0&0 \\ 0&1&0&0&0&0 \\ 1&0&0&0&0&0 \\ 0&0&0&1&0&0 \\ 0&0&0&0&1&0 \\ 0&0&0&0&0&1 \end{pmatrix} \tag{53}$$

where $T_{1,2,3}$ are defined in Supplementary Equations 48-50. $k_x,\ k_y,\ k_f$ thus play the role of Bloch wavevectors in characterizing the band structure. The solution to Supplementary Equation 51 defines the band structure for the three-dimensional lattice. Below we will refer to such a band structure as the "bulk" band structure.

Using the model developed above, we solve the band structure for the case of inversion-symmetry-breaking case and time-reversal breaking, as shown in Supplementary Figure 4.

Supplementary Note 4. Band Structure of a Stripe

In this section, we study the band structure and surface states of a lossless stripe of a honeycomb lattice of rings infinite in the spatial dimension x and the frequency dimension, and have N rows along y, as shown in Fig. 4a in the main text. The eigenstates are thus Bloch states with well-defined k_x and k_f. The wave function in a unit cell is characterized by $\{a_{j,n,0}, b_{j,n,0} | j = 1,2,3,4; n = 1...N\}$'s, where the first subscript j labels the section in each ring, and the second subscript n labels the n-th row along y. The last subscript 0 labels a particular resonance with resonant frequency ω_0 chosen as the zeroth order mode.

Supplementary Equations 48-50 hold for all n, with $k_y = 0$. The system of equations is:

$$\begin{pmatrix} a_{4,n,0} \\ b_{2,n,0} \end{pmatrix} = e^{i\frac{\delta}{3}} \begin{pmatrix} 1 & 0 \\ 0 & e^{i\frac{\sqrt{3}}{2}k_x a} \end{pmatrix} S(\gamma) \begin{pmatrix} e^{i\alpha\cos(\phi_A + k_f \Omega)} & 0 \\ 0 & e^{-i\frac{\sqrt{3}}{2}k_x a} \end{pmatrix} \begin{pmatrix} a_{2,n,0} \\ b_{1,n,0} \end{pmatrix},\ n = 1...N \tag{54}$$

$$\begin{pmatrix} a_{2,n,0} \\ b_{4,n,0} \end{pmatrix} = e^{i\frac{\delta}{3}} \begin{pmatrix} 1 & 0 \\ 0 & e^{-i\frac{\sqrt{3}}{2}k_x a} \end{pmatrix} S(\gamma) \begin{pmatrix} 1 & 0 \\ 0 & e^{i\frac{\sqrt{3}}{2}k_x a} e^{i\alpha\cos(\phi_B + k_f \Omega)} \end{pmatrix} \begin{pmatrix} a_{1,n,0} \\ b_{2,n,0} \end{pmatrix},\ n = 1...N \tag{55}$$

$$\begin{pmatrix} a_{1,n,0} \\ b_{1,n-1,0} \end{pmatrix} = e^{i\frac{\delta}{3}} S(\gamma) \begin{pmatrix} a_{4,n,0} \\ b_{4,n-1,0} \end{pmatrix},\ n = 2...N \tag{56}$$

Boundary conditions for the rows $n = 1$ and $n = N$ at the outer edges of the stripe are:

$$a_{1,1,0} = e^{i\frac{\delta}{3}} a_{4,1,0},\ b_{1,N,0} = e^{i\frac{\delta}{3}} b_{4,N,0} \tag{57}$$

These equations can again be written in the form of an eigenvalue problem:

$$T(k_x, k_f)u = e^{-i\frac{\delta}{3}}u \tag{58}$$

where $u = [a_{1/2/4,1...N,0}, b_{1/2/4,1...N,0}]^T$ and $T = T(k_x, k_f; \gamma, \alpha, \phi_A, \phi_B)$ captures the static and dynamic couplings and the boundary conditions. This eigenvalue problem defines a band structure with δ as a function of k_x and k_f. Below we refer to such a band structure as a projected band structure. The projected band structure contains the bulk states as defined by Supplementary Equation 51 projected onto the k_x and k_f plane, as well as additional surface states associated with the surface only. Comparisons between the band structure calculated using the waveguide amplitude mode and the tight binding model are shown in Supplementary Figures 5 and 6.

Supplementary Note 5. Surface State Transport

Now we consider a stripe of honeycomb lattice of lossy rings infinite in x, as shown in Fig. 4a in the main text. To excite the system, a set of continuous wave signals is sent into the rings on the edge of the stripe through waveguides coupled to the rings. The input signals have a spatial phase distribution in the x direction to probe states with Bloch wavevector k_x. Input signal into each waveguide consists of two frequencies separated by Ω with tunable phase delay, to allow probing of surface states associated with different Weyl points.

We solve for the steady state modal amplitudes of the driven lossy system. The modal amplitudes characterizing the system are $\{a_{j,n,m}, b_{j,n,m} | j = 1...4, n = 1...N, m = 1...M\}$'s, where j labels the section on a ring, n labels the row along y and m labels the frequency modes. Only M frequencies closest to the excitation frequencies are considered, since the loss in the rings results in an exponential decay in the amplitudes of modes as a function of their frequency difference from the excitation frequencies.

Under the excitation with a constant k_x and a small detuning δ from the resonant frequencies, Supplementary Equations 30-32 hold for all n and m:

$$\begin{pmatrix} a_{4,n,m} \\ b_{2,n,m} \end{pmatrix} = e^{i\frac{2m\pi+\delta}{3}} \begin{pmatrix} 1 & 0 \\ 0 & e^{-i(\frac{\sqrt{3}}{2}k_x a + \frac{2\pi m}{3})} \end{pmatrix} S(\gamma) \begin{pmatrix} 1 & 0 \\ 0 & e^{i(\frac{\sqrt{3}}{2}k_x a + \frac{2\pi m}{3})} \end{pmatrix} \begin{pmatrix} a_{3,n,m} \\ b_{1,n,m} \end{pmatrix},\ n = 1...N, m = 1...M \quad (59)$$

$$\begin{pmatrix} a_{2,n,m} \\ b_{4,n,m} \end{pmatrix} = e^{i\frac{2m\pi+\delta}{3}} \begin{pmatrix} 1 & 0 \\ 0 & e^{i(\frac{\sqrt{3}}{2}k_x a + \frac{2\pi m}{3})} \end{pmatrix} S(\gamma) \begin{pmatrix} 1 & 0 \\ 0 & e^{-i(\frac{\sqrt{3}}{2}k_x a + \frac{2\pi m}{3})} \end{pmatrix} \begin{pmatrix} a_{1,n,m} \\ b_{3,n,m} \end{pmatrix},\ n = 1...N, m = 1...M \quad (60)$$

$$\begin{pmatrix} a_{1,n,m} \\ b_{1,n-1,m} \end{pmatrix} = e^{i\frac{2m\pi+\delta}{3}} S(\gamma) \begin{pmatrix} a_{4,n,m} \\ b_{4,n-1,m} \end{pmatrix},\ n = 2...N, m = 1...M \quad (61)$$

Dynamic coupling is described by Supplementary Equation 33. With m truncated, and with the proper modulation phase choice shown in Supplementary Figure 2c, we obtain:

$$a_{3,n,m} = r \sum_{m'=1}^{M} i^{m'-m} J_{m'-m}(\alpha) e^{i(m'-m)\phi_A} a_{3,n,m'},\ n = 1...N, m = 1...M \quad (62)$$

$$b_{3,n,m} = r \sum_{m'=1}^{M} i^{m'-m} J_{m'-m}(\alpha) e^{i(m'-m)\phi_B} b_{3,n,m'},\ n = 1...N, m = 1...M \quad (63)$$

where $r \lesssim 1$ captures the round trip loss in the ring.

The boundary conditions for the rows $n = 1$ and $n = N$ at the outer edges of the stripe are:

$$b_{1,N,m} = e^{i\frac{2m\pi+\delta}{3}} b_{4,N,m},\ a_{1,1,m} = e^{i\frac{2m\pi+\delta}{3}} a_{4,1,m} \quad (64)$$

for all the modes except the ones that directly couple to inputs.

To excite the surface states on the top edge of the stripe, consider inputs to the row $n = 1$ for the A sublattice, using two frequencies $\omega_{M_0} + \delta$ and $\omega_{M_0+1} + \delta$, where ω_{M_0} and ω_{M_0+1} are the frequencies of the M_0-th and (M_0+1)-th resonant modes with $\omega_{M_0+1} - \omega_{M_0} = \Omega$, and δ is a small detuning. The amplitudes of the excitation at these two frequencies are S_{M_0} and S_{M_0+1}, respectively. The boundary conditions are modified for the M_0-th and (M_0+1)-th modes as:

$$e^{-i\frac{2M_0\pi+\delta}{6}} a_{1,1,M_0} = e^{i\frac{2M_0\pi+\delta}{6}} \sqrt{1-\eta^2} a_{4,1,M_0} + i\eta S_{M_0} \quad (65)$$

$$e^{-i\frac{2(M_0+1)\pi+\delta}{6}} a_{1,1,M_0+1} = e^{i\frac{2(M_0+1)\pi+\delta}{6}} \sqrt{1-\eta^2} a_{4,1,M_0+1} + i\eta S_{M_0+1} \quad (66)$$

where η is the coupling between the input waveguides and the rings. By choosing the relative phase of the inputs S_{M_0} and S_{M_0+1}, we can excite certain k_f modes selectively.

Similarly, to excite surface states on the bottom edge of the stripe, we can instead impose the following boundary conditions at the row $n = N$ for the B sublattice for the M_0-th and (M_0+1)-th modes:

$$e^{-i\frac{2M_0\pi+\delta}{6}} b_{1,N,M_0} = e^{i\frac{2M_0\pi+\delta}{6}} \sqrt{1-\eta^2} b_{4,N,M_0} + i\eta S_{M_0} \tag{67}$$

$$e^{-i\frac{2(M_0+1)\pi+\delta}{6}} b_{1,N,M_0+1} = e^{i\frac{2(M_0+1)\pi+\delta}{6}} \sqrt{1-\eta^2} b_{4,N,M_0+1} + i\eta S_{M_0+1} \tag{68}$$

Next, we combine Supplementary Equations 59-66 into a linear matrix equation to solve for the steady state modal amplitudes $\{a_{j,n,m}, b_{j,n,m} | j = 1...4, n = 1...N, m = 1...M\}$'s with inputs to the top edge:

$$u = e^{i\frac{\delta}{3}} T(k_x) u + (e^{i\frac{\delta}{6}} i\eta) w \implies u = [I - e^{i\frac{\delta}{3}} T(k_x)]^{-1} (e^{i\frac{\delta}{6}} i\eta) w \tag{69}$$

Here $u = [..., a_{1,n,m}, b_{1,n,m}, a_{2,n,m}, b_{2,n,m}, ...]^T$ is a vector of length $8NM$, and $T = T(k_x; \gamma, \alpha, \phi_A, \phi_B, r, \eta)$ represents the static and dynamic couplings and the boundary conditions in Supplementary Equations 59-66. w represents the input amplitudes in Supplementary Equations 65 and 66. w is a vector of mostly 0, except at $a_{1,1,M_0}$ and $a_{1,1,M_0+1}$ which have values $e^{iM_0\pi/3} S_{M_0}$ and $e^{i(M_0+1)\pi/3} S_{M_0+1}$, respectively.

Similarly, with input to the bottom edge, we combine Supplementary Equations 59-64 and 67-68 into a matrix equation which has the same form as Supplementary Equation 69. The expressions for $T(k_x)$ and w are different due to different boundary conditions at the input modes. In this case w has values $e^{iM_0\pi/3} S_{M_0}$ and $e^{i(M_0+1)\pi/3} S_{M_0+1}$ at b_{1,N,M_0} and b_{1,N,M_0+1}, respectively, and 0's elsewhere.

For the case of inversion symmetry breaking, the Weyl points are near $\delta = 0$, $k_x = 2\pi/3\sqrt{3}a$ or $4\pi/3\sqrt{3}a$, and $k_f = \pm\pi/2\Omega$. Input with $\delta = 0$, $k_x = \pi/\sqrt{3}a$ and a relative phase of $S_{M_0+1}/S_{M_0} = \pm i$ thus selectively couples to the surface states at the midpoint of the surface state arcs near $k_f = \pm\pi/2\Omega$, respectively. In the presence of input to the top row of rings, steady state modal amplitudes are plotted in Figs. 4f,g of the main text. Supplementary Figures 5e,f show the steady states with input to the bottom edge. The resulting modal amplitudes are confined to the rings on the respective edges, demonstrating the existence of surface states. With a choice of input that selectively couples to $k_f = -\pi/2\Omega$, the system exhibits one-way frequency down conversion on the bottom edge, and one-way frequency up conversion on the top edge [Fig. 4f in the main text]. Frequency conversion in the opposite direction is observed with a choice of input that selectively couples to $k_f = -\pi/2\Omega$. This proves that the surface states are one-way, and have different chirality near $k_f = \pm\pi/2\Omega$.

Supplementary Figures 6l,m show the case of time-reversal symmetry breaking with $\phi_A = -\phi_B = \pi/3$ and input to the top edge. In this case the Weyl points near $k_f = 0$ and $k_f = \pi/\Omega$ have frequencies close to $\delta/\gamma = \pm 1$, respectively. To excite the modes at the midpoint of the surface state arcs near $k_f = 0$, we choose $\delta = \gamma$, $k_x = \pi/\sqrt{3}a$ and $S_{M_0}/S_{M_0+1} = 1$. This leads to one-way frequency up-conversion, as shown in Supplementary Figure 6l. To excite the modes at the midpoint of the surface state arcs near $k_f = \pi/\Omega$, we choose $\delta = -\gamma$, $k_x = \pi/\sqrt{3}a$ and $S_{M_0}/S_{M_0+1} = -1$. This leads to one-way frequency down-conversion, as shown in Supplementary Figure 6m. These results again confirm the existence and one-way frequency conversion of surface states.

For the time-reversal symmetry breaking case, the excitation is not as well confined to the surface as in the case of inversion symmetry breaking. For the case of time-reversal symmetry breaking shown in Supplementary Figure 6, the third row of $y(i)$, corresponding to the second row of A resonators, has non-zero intensity. The reason is the existence of bulk states at the same frequencies and k_x as the surface arcs. For example in Supplementary Figure 6l, $k_x = \pi/\sqrt{3}a$, $\delta = \gamma$ and $S_{M_0+1}/S_{M_0} = 1$ is chosen to maximize excitation of the surface states near the midpoint of the pair of Weyl points at $k_f = 0$. However, there are bulk states near $k_f = \frac{\pi}{\Omega}$ at the same frequency and k_x. Although the excitation phase eliminates coupling to states with exactly $k_f = \frac{\pi}{\Omega}$, coupling to other k_f states are not completely suppressed. Some of the excitation leaks through these bulk states. In comparison, for the case of inversion symmetry breaking, the Weyl points are nearly degenerate. For $\delta \approx 0$ the bulk state density is very low, and is absent for most of the range of $2\pi/3\sqrt{3}a < k_x < 4\pi/3\sqrt{3}a$ that supports surface states. This results in better edge confinement shown in Supplementary Figures 5e,f and Figs. 4e,f in the main text.

Supplementary Note 6. Design of an experimental system

To facilitate comparison between different systems, we normalize the frequency units with respect to the free spectral range (FSR) of the resonators. With this normalization, the inter-ring coupling constant $t_{xy} = \gamma\times$FSR, nearest-neighbour coupling is $t_f = J_1(\alpha)\times$FSR, and the internal loss rate is $\kappa_{\text{in}} = -\ln(r)\times$FSR, where r is round-trip amplitude transmission in the ring. To preserve the validity of rotating wave approximation, the coupling strength and the modulation frequency has to satisfy [3] $t_{xy}, t_f \leq \Omega/6 \approx$FSR, where $\Omega = 2\pi\times$FSR is the modulation frequency. In order to observe one-way frequency conversion in edge state before loss become dominant, the system also need to satisfy $t_{xy}, t_f \gg \kappa_{\text{in}}$.

Here we consider a concrete example using silicon ring resonator on silicon-on-insulator platform. Supplementary Table 1 lists parameters for two possible designs using carrier-depletion modulator in silicon rib waveguide. In this system, the RF modulation frequency is limited to a few tens of GHz [6], which coincides with the free spectral range of the ring. Consequently, the length of the waveguide forming the ring needs to be on the order of one millimetre. For a modulation frequency of 26 GHz, a simple ring with 445 µm radius is required, as experimentally demonstrated in [4]. As shown in Supplementary Figure 8, a 4×3 array of ring resonators are already sufficient for demonstrating the Fermi arc surface states, thus the overall dimension of the ring array is 3×4 mm^2, which fits well on a single chip. On this length scale, however, fabrication-related variation may still lead to large resonant mismatch between rings [7–9]. Since the ring size is relatively large, and there are only 12 resonators, it is possible to use heaters to thermally aligned the resonances. In addition, one can also reduce the size of the resonator by folding the waveguide [10], e.g. as shown in Supplementary Figure 9, so that the resonator can be enclosed in a 150 µm radius area, 1/3 of its original size. With this, the whole array can be shrunk down to 1 mm in size. The size can be further reduced by increasing the RF modulation frequency to 50 GHz, which has been achieved in silicon carrier-depletion modulator [6] with careful optimization.

Index modulation in silicon is based on the plasma dispersion effect, which is intrinsically lossy. Carrier-depletion based modulation, which changes the width of the depletion region in a pn junction formed inside the waveguide, results in lower optical losses as compared to modulation schemes based on carrier injection. Loss due to carrier absorption is proportional to the carrier density, while coupling strength between adjacent modes separated in frequency by one FSR is proportional to both the carrier density and the modulation voltage. Coupling strength of 0.5 mm^{-1} accompanied by absorption loss of less than 1 dB mm^{-1} with 3 V modulation voltage has been demonstrated in silicon carrier-depletion modulator based Mach-Zehnder interferometer [5, 6]. With such coupling and absorption loss, the requirements $t_{xy}, t_f \leq$FSR and $t_{xy}, t_f \gg \kappa_{\text{in}}$ can be satisfied, with specific examples shown in Supplementary Table 1. For a FSR=26 GHz ring with 1/3 of its circumference modulated, one may obtain a coupling constant $t_f = 8$ GHz $\approx$ FSR/3, and a loss rate $\kappa_{\text{in}} = 2.7$ GHz $\approx t_f/3$. An intrinsic quality factor (including waveguide loss and carrier absorption) of 2×10^5 is required, which is well within the reach of high Q passive silicon ring resonators [11, 12], but still a few times higher than that demonstrated for active rings [4]. However, carrier-depletion based silicon ring modulator has not been well-optimized for the modulation frequency matching to the free-spectral range, thus careful design of doping concentration and waveguide cross-section [5] could in principle achieve the desirable quality factor in active ring modulators.

Using the coupling constant $t_f = 8$ GHz and loss $\kappa_{in} = 2.7$ GHz from the 26 GHz FSR example, we simulate the excitation of one-way frequency conversion in edge states on a 4×3 array of ring resonators with inversion-symmetry-breaking modulation phase. The result is shown in Supplementary Figure 8. We observe a good confinement to the edge, and one-way frequency conversion propagating over 6 cavity modes before the intensity is reduced below 1/10.

In our simulation shown in Supplementary Figure 8, the loss is large enough that the fields do not propagate to the boundary along the frequency axis, which may be caused by a sudden increase of refractive index dispersion or absorption. On the other hand, if the loss is low enough, and there is a sharp boundary along the frequency axis, then upon reaching the boundary, there is always scattering into the bulk states since the bulk is not gapped. In practice, such scattering into the bulk state may be small due to the small bulk density of states near the frequency range of the Weyl points. For system without inversion symmetry symmetry (Supplementary Figure 5), such a boundary also creates back-scattering since the same edge supports counter-propagating edge states at the same frequency. For system without time-reversal symmetry (Supplementary Figure 6), back-scattering does not occur since there is no counter-propagating edge state with the same frequency at the same edge as the forward propagating state.

While we use carrier-depletion modulation based silicon ring resonator as an example due to their compactness for on-chip integration and popularity as an integrated photonics platform, $LiNbO_3$ [13–15] and polymer [16] modulators may have high optical Q, stronger and intrinsically lossless electro-optic effect and higher modulation bandwidth, making them attractive platforms for demonstrating our proposed scheme for achieving Weyl point in synthetic frequency dimension as well.

Supplementary References

[1] Yuan, L., Yu, S. & Fan, S. Photonic gauge potential in a system with a synthetic frequency dimension. *Opt. Lett.* **41**, 741-744 (2016).

[2] Hafezi, M., Mittal, S., Fan, J., Migdall, A. & Taylor, J. M. Imaging topological edge states in silicon photonics. *Nature Photon.* **7**, 1001-1005 (2013).

[3] Yuan, L. & Fan, S. Topologically nontrivial Floquet band structure in a system undergoing photonic transitions in the ultrastrong-coupling regime. *Phys. Rev. A* **92**, 053822 (2015).

[4] Tzuang, L. D., Soltani, M., Lee, Y. H. D. & Lipson, M. High RF carrier frequency modulation in silicon resonators by coupling adjacent free-spectral-range modes. *Opt. Lett.* **39**, 1799-1802 (2014).

[5] Xiao, X. *et al.* High-speed, low-loss silicon Mach-Zehnder modulators with doping optimization. *Opt. Express* **21**, 4116-4125 (2013).

[6] Reed, G. T. *et al.* Recent breakthroughs in carrier depletion based silicon optical modulators. *Nanophotonics* **3**, 229-245 (2014).

[7] Chrostowski, L. *et al.* Impact of fabrication non-uniformity on chip-scale silicon photonic integrated circuits. *Optical Fiber Communication Conference, Th2A.37* (2014). doi:10.1109/OFC.2014.6886717

[8] Zortman, W. A., Trotter, D. C. & Watts, R. M. Silicon photonics manufacturing. *Opt. Express* **18**, 23598-23607 (2010).

[9] Sun, J., Timurdogan, E., Yaacobi, A., Hosseini, E. S. & Watts, M. R. Large-scale nanophotonic phased array. *Nature* **493**, 195-199 (2013).

[10] Aboketaf, A. A., Elshaari, A. W. & Preble, S. F. Optical time division multiplexer on silicon chip. *Opt. Express* **18**, 13529-13535 (2010).

[11] Griffith, A., Cardenas, J., Poitras, C. B. & Lipson, M. High quality factor and high confinement silicon resonators using etchless process. *Opt. Express* **20**, 21341-21345 (2012).

[12] Xiao, S., Khan, M. H., Shen, H. & Qi, M. Compact silicon microring resonators with ultra-low propagation loss in the C band. *Opt. Express* **15**, 14467-14475 (2007).

[13] Cohen, D. A., Hossein-Zadeh, M. & Levi, A. F. J. High- Q microphotonic electro-optic modulator. *Solid State Electron.* **45**, 1577-1589 (2001).

[14] Ilchenko, V. S., Savchenkov, A. A., Matsko, A. B. & Maleki, L. Whispering-gallery-mode electro-optic modulator and photonic microwave receiver. *J. Opt. Soc. Am. B-Optical Phys.* **20**, 333-342 (2003).

[15] Ilchenko, V. S., Savchenkov, A. A., Matsko, A. B. & Maleki, L. Nonlinear optics and crystalline whispering gallery mode cavities. *Phys. Rev. Lett.* **92**, 43903 (2004).

[16] Bortnik, B. *et al.* Electrooptic polymer ring resonator modulation up to 165 GHz. *IEEE J. Sel. Top. Quantum Electron.* **13**, 104-109 (2007).
